# The Human Resources for Health Effort Index: a tool to assess and inform Strategic Health Workforce Investments

**DOI:** 10.1186/s12960-017-0223-2

**Published:** 2017-07-19

**Authors:** Alfredo L. Fort, Rachel Deussom, Randi Burlew, Kate Gilroy, David Nelson

**Affiliations:** 1 IMA World Health, Washington, DC USA; 2 FHI360, Washington, DC USA; 3 Philliber Research and Evaluation, Accord, NY USA; 4 Maternal and Child Survival Program, Washington, DC USA; 5 IntraHealth International, Chapel Hill, NC USA

**Keywords:** Human resources for health, Health systems strengthening, Index, Health workforce

## Abstract

**Background:**

Despite its importance, the field of human resources for health (HRH) has lagged in developing methods to measure its status and progress in low- and middle-income countries suffering a workforce crisis. Measures of professional health worker densities and distribution are purely numerical, unreliable, and do not represent the full spectrum of workers providing health services. To provide more information on the multi-dimensional characteristics of human resources for health, in 2013–2014, the global USAID-funded Capacity*Plus* project, led by IntraHealth International, developed and tested a 79-item HRH Effort Index modeled after the widely used Family Planning Effort Index.

**Methods:**

The index includes seven recognized HRH dimensions: Leadership and Advocacy; Policy and Governance; Finance; Education and Training; Recruitment, Distribution, and Retention; Human Resources Management; and Monitoring, Evaluation, and Information Systems. Each item is scored from 1 to 10 and scores are averaged with equal weights for each dimension and overall. The questionnaire is applied to knowledgeable informants from public, nongovernmental organization, and private sectors in each country. A pilot test among 49 respondents in Kenya and Nigeria provided useful information to improve, combine, and streamline questions. Capacity*Plus* applied the revised 50-item questionnaire in 2015 in Burkina Faso, Dominican Republic, Ghana, and Mali, among 92 respondents. Additionally, the index was applied subnationally in the Dominican Republic (16 respondents) and in a consensus-building meeting in Mali (43 respondents) after the national application.

**Results:**

The results revealed a range of scores between 3.7 and 6.2 across dimensions, for overall scores between 4.8 and 5.5. Dimensions with lower scores included Recruitment, Distribution, and Retention, while Leadership and Advocacy had higher scores.

**Conclusions:**

The tool proved to be well understood and provided key qualitative information on the health workforce to assist in health systems strengthening. It is expected that subsequent applications should provide more information for comparison purposes, to refine aspects of the questionnaire and to correlate scores with measures of service outputs and outcomes.

## Background

Human resources for health (HRH) is an essential component of the World Health Organization (WHO)’s health system building blocks, and crucial for improving health outcomes [1, 2]. Yet less attention has been given to HRH compared to other building blocks, and thus less accurate and complete data have been generated to inform effective HRH policies, strategies, and practices. The WHO’s Workforce 2030 global strategy on HRH and the 2016 High-Level Commission on Health Employment and Economic Growth’s recommendations on investing in the health workforce call for improved health workforce data and analytics and accelerated country progress on sharing HRH data and reporting annually on core indicators [3, 4].

The simplest and most direct way of looking at the effect of HRH on health services is quantifying health worker presence. The WHO has been using the concept of the *density* of health workers in respect to population. Its seminal 2006 World Health Report devoted to HRH used a correlation of the density of doctors, nurses, and midwives per 1000 population to the coverage of skilled birth attendance (SBA) to derive a threshold of 2.28 health workers (later standardized to 22.8 per 10 000 population) to achieve a minimum desired coverage of 80% SBA [5].

In a more recent publication, a higher threshold of 34.5 skilled health professionals per 10 000 population was determined based on a model of population’s access to an expanded health benefits package (conducted by the International Labour Organization). An even higher threshold of 59.4 was added after a joint exercise between WHO and the US Agency for International Development (USAID) found this was the rate (achieved by Mexico) needed to attain a reduction in the maternal mortality ratio to 50 per 100 000 live births by 2035 [6].

However, another analysis found that countries with similar health worker densities can have dramatically different health outcomes, suggesting that HRH strengthening is not as simple as having sufficient overall numbers of health workers in the country [7]. A similar indicator is the Health Worker Reach Index, which incorporates the WHO density indicator and adds measures of access to and use of services provided by health workers [8].

There are a number of limitations to these quantitative indicators. First, estimates are nonexistent or inaccurate for many low-income countries. For example, in a 2013 WHO report, 17 of the 57 countries identified as having low HRH densities in 2006 did not have any data point in the previous 5 years [6]. Another limitation is that indicators exclude certain cadres of health workers, such as auxiliary and community health workers, who are vastly responsible for delivering health care in many countries, especially in remote areas.

However, a bigger limitation is that these indicators do not provide information on the more qualitative aspects and processes of HRH. For example, there is hardly any information on the capacity of HRH offices and leaders, sufficiency of HRH budgets in ministries, links between preservice education and professional development of the workforce, and so on. This lack of information prevents or severely constrains countries and donors from identifying specific program and policy efforts to address gaps in HRH, and to track progress over time. Further, by not knowing the importance of inputs, processes, and even outputs on HRH interventions, there is little understanding of their relationships to service supply and use, and potentially on health outcomes. Such information could guide and help countries make smarter investments with existing funds.

Within the HRH arena, there are a number of dimensions that interface with each other to result in an “improved health workforce.” WHO’s Global Health Workforce Alliance (GHWA) and USAID developed the HRH Action Framework (HAF) [9], which has six “action fields”: Policy, Finance, Education, Leadership, Partnership, and Human Resources Management Systems. These fields are encircled by four “phases” or action cycles: Situational Analysis, Planning, Implementation, and Monitoring and Evaluation, all of which influence health workforce output. In turn, the framework posits that an improved workforce, together with other health system components and country-specific contexts, will lead to equitable, effective, efficient, and quality provision of health services that will result in improved health outcomes.

The HAF serves as a starting point for discussion about the role of each dimension. For example, no one can deny the role of good management of human resources in the ultimate delivery of health care services [10]. However, less is known about the status and inputs in countries that influence other dimensions, such as leadership, finance, or policy.

With the objectives of developing a tool to obtain information on critical dimensions of HRH, and applying it in countries investing in this area, the USAID-funded global Capacity*Plus* project, led by IntraHealth International, developed an HRH Effort Index (available at https://www.intrahealth.org/resources/human-resources-health-effort-index). The index is modeled after the successful and widely used Family Planning Effort Index (FPE), developed in the mid-1970s to measure the level of effort by governments to implement national family planning programs [11]. The FPE was tested, amended, and has subsequently been used in more than 80 countries, including at provincial levels, to relate its findings to outcome measures such as fertility and contraception [12–14]. The FPE asked 10–15 key informants to rate, on a scale of 1 to 10, the status or level of effort on 30 key FP program areas, under four components: Policies, Services, Evaluation, and Method Access. Despite initial hesitation and criticism, the index proved consistent and robust as more applications occurred and summaries were obtained for countries, regions, and globally [15]. Another similar and more recent index that has also met wide acceptance and use is the AIDS Program Effort Index (API) [16–18].

## Methods

Capacity*Plus* conceptualized the HRH Effort Index in 2013 in consultation with creators and users of indices, and informed by literature reviews. With the participation of an advisory group, a first draft of the index’s survey tool was completed in 2014, with 79 items distributed over seven recognized HRH dimensions: (1) Leadership and Advocacy, (2) Policy and Governance, (3) Finance, (4) Education and Training, (5) Recruitment, Distribution, and Retention, (6) Human Resources Management, and (7) Monitoring, Evaluation, and Information Systems. Each item is scored on a scale of 1 to 10, from weaker to stronger. All items are averaged to produce a score for each dimension and in turn all dimensions are averaged into an overall score. All items and dimensions are weighted equally.

Capacity*Plus* pilot-tested the index among 49 respondents in Kenya (22) and Nigeria (27) in May–June 2014. This first application yielded differences in total scoring between the two countries (Kenya 5.7 and Nigeria 4.2) as well as variations in partial scoring [19]. The pilot application provided feedback from respondents, the advisory group, and consultants, to streamline the questionnaire for future applications. Some questions were deemed repetitive and others too complex. An inter-correlation matrix constructed with the combined data set identified variables that seemed unique (i.e., measured a discrete aspect of HRH efforts) and others that were highly correlated, thus probably measuring similar HRH efforts. With this information, the original tool was revised and the number of items reduced to 50, while keeping the original seven dimensions. The advisory group also recommended application of the index in different contexts, such as at subnational levels as well as in a group meeting to elicit buy-in through a consensus exercise.

After final revision in March 2015, the HRH Effort Index was applied at the national level in Burkina Faso, the Dominican Republic (DR), Ghana, and Mali, from April to September 2015, as well as at a subnational level in the DR and as a consensus application in Mali. As with other indices, sampling was necessarily purposive for these applications given the need to identify knowledgeable respondents. However, every effort was made to ensure wide representation of respondents from different sectors.

Three formats were employed to administer the index, as deemed contextually appropriate: manually filling out hard copy forms handled by a consultant, electronic completion via PDF, and an Internet-based modality (SurveyMonkey). Local consultants were hired or project assistance provided locally to make contact with respondents and ensure they understood all instructions. As with the pilot application, the index was administered to a wide mix of public and private HRH and health systems experts representing different groups of civil society.

## Results

### Respondents

There was a large variation in the characteristics of respondents, depending on the country of application. In the three African countries, the majority of respondents were male, while in the two applications in the Dominican Republic, the majority were women. Overall, nearly 6 out of 10 respondents were male. This distribution mirrors the relative gender balance among high-level officers in the countries investigated.

Regarding employer, in the three African countries the majority of respondents worked for the national government (Ministry of Health (MOH) or other entity), while in the two applications in the Dominican Republic, there was more variety among institutions, including health facilities, health professional schools or universities, professional associations, and other civil society organizations. Overall, 7 out of 10 respondents worked in national or local governments. This distribution is logical given the interest in exploring overarching HRH aspects in each country such as leadership, policy, governance, and resource distribution.

As expected, the majority of respondents were managers or directors in their respective organizations. In Burkina Faso as in Mali a respectable minority were mid-level officers and/or consultants.

In total, the HRH Effort Index was applied to about 100 individuals, who provided valuable responses for its different dimensions (Table 1) that are analyzed in subsequent sections.

**Table 1:** Number and characteristics of respondents to the HRH Effort Index in each country

Characteristic	Burkina Faso		Dominican Republic—national		Dominican Republic—subnational		Ghana		Mali		Total	
	*N*	Percentage	*N*	Percentage	*N*	Percentage	*N*	Percentage	*N*	Percentage	*N*	Percentage
Sex												
Male	14	74%	11	40%	6	37%	12	66%	19	70%	62	57%
Female	5	26%	17	60%	10	63%	6	34%	8	30%	46	43%
* N*	19	100%	28	100%	16	100%	18	100%	27	100%	108	100%
Employer												
National government	15	79%	12	43%	7	32%	10	56%	18	67%	62	65%
State/local government	–	–	2	7%	2	10%	4	22%	1	4%	9	9%
Health facility/clinical service	–	–	5	18%	4	18%	–	–	1	4%	10	11%
Health professional school/university /research organization	3	16%	4	14%	3	14%	1	5%	1	4%	9	9%
Professional association	–	–	3	11%	1	5%	–	–	2	7%	6	6%
Nongovernmental/faith-based organization/international organization	1	5%	1	4%	1	5%	1	5%	–	–	3	3%
Private/for-profit organization	–	–	1	4%	1	5%	2	12%	1	4%	5	5%
Civil society	–	–	–	–	3	14%	–	–	3	11%	6	6%
* N*	19	100%	28	100%	22	100%	18	100%	27	100%	95	100%
Position												
Manager/director/CEO	10	53%	24	96%	16	100%	17	94%	20	74%	87	81%
Officer/consultant	5	26%	1	4%	–	–	–	–	4	14%	10	9%
Clinician	–	–	–	–	–	–	–	–	1	4%	1	1%
Academia/researcher	3	16%	–	–	–	–	1	6%	–	–	4	4%
Other	1	5%	–	–	–	–	–	–	2	7%	3	3%
Total^a^	19	100%	28	100%	16	100%	18	100%	27	100%	108	100%

^a^Some «*N*» may not sum up to the total due to missing responses

### Leadership and Advocacy

The Leadership and Advocacy dimension comprises five questions that assess the strength and presence of high-level leadership and its capacity to advocate on behalf of the health workforce in the country. See Table 2 for a breakdown of scores per item by country.

**Table 2:** Number of respondents and their scores for the Leadership and Advocacy dimension of the HRH Effort Index

Item #	I. Leadership and Advocacy	Burkina Faso		Dominican Republic—national		Dominican Republic—subnational		Ghana		Mali		Total	
		*N*	Score	*N*	Score	*N*	Score	*N*	Score	*N*	Score	*N*	Score
1.	Human resources for health (HRH) prominence within the Ministry of Health	19	5.9	25	6.1	15	6.5	20	7.0	26	6.2	105	6.3
2.	Political support for HRH	19	4.3	25	5.8	16	5.8	20	5.8	25	4.7	105	5.3
3.	Influence of HRH leaders or champions	19	5.2	24	5.5	16	5.3	20	5.0	26	5.4	105	5.3
4.	Strength of an HRH observatory/stakeholder/technical leadership group	18	5.1	23	5.7	14	7.0	20	4.9	24	6.6	99	5.9
5.	Media coverage for HRH	19	5.0	24	5.4	16	6.3	20	6.6	27	4.8	106	5.6
Average respondents and scores		19	5.1	24	5.7	15	6.2	20	5.8	26	5.5	104	5.7

Most respondents scored the items in this dimension at mid-point or above. In particular, prominence of HRH within the MOH received high marks in all countries. Political support for HRH scored lower (4.3) in Burkina Faso and Mali (4.7). Respondents in Mali indicated that there was an HRH directorate, but that it lacked financial support from the government. Overall, respondents had a positive view of Leadership and Advocacy, with an average score of 5.7.

### Policy and Governance

The Policy and Governance dimension includes six items that assess how well the country has developed policies and strategies to support, develop, and manage the health workforce. See Table 3 for scoring of individual items per country application.

**Table 3:** Number of respondents and their scores for the Policy and Governance dimension of the HRH Effort Index

Item#	II. Policy and Governance	Burkina Faso		Dominican Republic—national		Dominican Republic—subnational		Ghana			Mali	Total	
		*N*	Score	*N*	Score	*N*	Score	*N*	Score	*N*	Score	*N*	Score
1.	National HRH plan	19	5.6	24	5.2	14	5.9	19	6.5	26	6.8	102	6.0
2.	Evidence-based national HRH strategies	16	5.6	23	4.9	14	4.7	20	6.0	23	6.7	96	5.6
3.	Recognized and defined health worker cadres and scopes of practice	18	5.7	23	5.0	15	6.1	20	6.2	25	6.5	101	5.9
4.	Inclusion of nongovernmental actors in the national HRH plan	18	4.8	24	4.5	15	5.4	20	6.1	24	5.2	101	5.2
5.	Health workforce remuneration	19	5.0	25	4.0	16	3.7	20	6.6	23	4.7	103	4.8
6.	Gender and diversity inclusion in the national HRH plan	19	6.5	23	5.5	16	5.8	20	6.1	24	5.1	102	5.8
Average respondents and scores		18	5.5	24	4.8	15	5.3	20	6.2	24	5.9	101	5.5

While generating an overall score above the mid-point, this dimension scored low on health workforce remuneration, which was only seen in a favorable light in Ghana (score of 6.6) and as average in Burkina Faso. It received one of the lowest marks of the index in the subnational application in the Dominican Republic (3.7) and a similarly low mark in the DR national application (4.0). A respondent in the Dominican Republic stated that salaries were comparatively lower for nurses “in relation to their workload and teaching responsibilities.”

Another component with relatively low scores is the inclusion of nongovernmental actors in the national HRH plan. In Ghana a respondent indicated that for HRH there is “little or no civil society participation.” In the national applications in Burkina Faso and the Dominican Republic, this component received scores below the mid-point.

### Finance

The Finance dimension of the index includes seven items that assess the extent to which the country has adequate funding to support the health workforce. See Table 4 for scores by item and country.

**Table 4:** Number of respondents and their scores for the Finance dimension of the HRH Effort Index

Item #	III. Finance	Burkina Faso		Dominican Republic—national		Dominican Republic—subnational		Ghana		Mali		Total	
		*N*	Score	*N*	Score	*N*	Score	*N*	Score	*N*	Score	*N*	Score
1.	Costed national HRH plan	18	4.9	22	4.3	16	4.7	17	5.4	20	4.7	93	4.8
2.	Domestic funding of the national HRH plan	16	4.7	19	5.1	12	3.9	18	5.2	22	3.8	87	4.5
3.	Funding for producing adequate numbers of qualified health workers	19	4.6	23	4.0	14	5.0	17	5.2	19	4.3	92	4.6
4.	Access to and availability of funding for tuition for preservice education	19	3.8	23	5.0	15	4.7	18	5.3	26	3.7	101	4.5
5.	Funding for in-service training and continuing professional development	19	4.2	23	4.3	15	4.9	19	3.7	26	4.2	102	4.3
6.	Government payroll system	19	7.0	25	7.0	16	6.3	20	6.8	24	7.5	104	6.9
7.	Funding for human resources information systems (HRIS)	17	3.9	23	4.7	15	4.9	17	3.4	20	5.2	92	4.4
Average respondents and scores		18	4.8	23	4.9	15	4.9	18	5.0	22	4.8	96	4.9

While it averaged near the mid-point, this dimension saw wide variation according to specific items. For example, Ghana assigned the lowest score to funding for human resources information systems (3.4), an issue also shared by Burkina Faso. Mali gave a low score for access to and availability of funding for tuition for preservice education, with a similar low mark given by Burkina Faso. Mali also scored low (3.8) for domestic funding of the national HRH plan, similarly scored by the subnational application in the Dominican Republic.

Contrastingly, the item of government payroll system received very high ratings in all countries, with Mali assigning it one of the highest scores seen at 7.5. It is important to note that the overall high score obtained for this item compensates the overall lower scores (below the mid-point) that countries assigned to almost all other items in this dimension.

### Education and Training

The Education and Training dimension of the index is comprised of 10 items designed to assess the ability of the country’s education system to produce and adequately train health workers (i.e., the respective strengths of preservice education, in-service training, and continuing professional development). See Table 5 for a list of scores by item and country.

**Table 5:** Number of respondents and their scores for the Education and Training dimension of the HRH Effort Index

Item #	IV. Education and Training	Burkina Faso		Dominican Republic—national		Dominican Republic—subnational		Ghana		Mali		Total	
		*N*	Score	*N*	Score	*N*	Score	*N*	Score	*N*	Score	*N*	Score
1.	Health workforce education strategy	15	5.3	25	4.8	14	4.6	19	4.9	26	5.2	99	5.0
2.	Gender in preservice education (PSE) policy	19	5.6	23	5.2	15	5.8	19	5.1	26	4.8	102	5.3
3.	Quality preservice health institutions and education	17	5.8	22	5.4	15	5.7	19	7.1	26	6.1	99	6.0
4.	Adequate faculty for PSE institutions	18	6.5	22	5.7	14	6.4	19	5.9	25	5.7	98	6.0
5.	Diversity in student recruitment	19	3.7	18	4.9	15	5.0	19	6.1	25	4.7	96	4.9
6.	Preservice education student tracking	18	5.7	21	4.7	15	5.0	17	5.1	25	4.9	96	5.1
7.	High health worker graduation and certification rates (low dropout rates)	19	7.0	19	5.7	14	6.5	18	6.0	23	5.9	93	6.2
8.	Provision of career support to preservice education graduates	19	4.5	21	4.2	15	4.2	19	4.5	25	4.4	99	4.4
9.	Provision of relevant in-service training to health workers	19	5.7	23	4.6	15	4.9	19	4.6	24	5.2	100	5.0
10.	Links between in-service training and certification/relicensure	14	5.0	19	4.3	12	5.3	17	6.4	22	5.1	84	5.2
Average respondents and scores		18	5.5	21	5.0	14	5.3	19	5.6	25	5.2	97	5.3

While this dimension is scored at mid-point overall, it revealed some interesting variations in the ratings of items. All countries scored high on having adequate faculty for preservice education institutions. For example, Burkinabe respondents claimed that “students from neighboring countries come to Burkina to study.” Two other items with high overall marks were high health worker graduation and certification rates (low dropout rates), which respondents in Burkina Faso granted a very high rating of 7, and quality preservice health institutions and education, mostly because of a very high score from respondents in Ghana. However, provision of career support to preservice education graduates received an overall low score. Here respondents in the Dominican Republic subnational application noted that pregnant women were “stigmatized” in schools and single mothers faced difficulties continuing their studies.

### Recruitment, Distribution, and Retention

The fifth dimension of the index—Recruitment, Distribution, and Retention—is made up of five items designed to assess the extent to which health workers are dispersed across the country, such that the needs of all members of the population (i.e., in both rural and urban areas) are met. See Table 6 for individual items and their scores by country.

**Table 6:** Number of respondents and their scores for the Recruitment, Distribution, and Retention dimension of the HRH Effort Index

Item #	V. Recruitment, Distribution, and Retention	Burkina Faso		Dominican Republic—national		Dominican Republic—subnational		Ghana		Mali		Total	
		*N*	Score	*N*	Score	*N*	Score	*N*	Score	*N*	Score	*N*	Score
1.	Health workforce analysis of shortages and labor market dynamics	17	4.5	23	4.0	15	4.3	19	5.8	25	4.9	99	4.7
2.	Absorption of preservice education graduates	18	5.3	24	3.5	16	4.1	19	5.5	26	3.7	103	4.4
3.	Effectiveness of health workforce recruitment strategies	17	5.7	24	3.8	16	3.9	19	6.0	25	4.6	101	4.8
4.	Effectiveness of health workforce deployment and distribution strategies	19	4.6	23	3.5	16	3.8	19	5.3	26	3.7	103	4.2
5.	Effectiveness of health worker retention strategies	19	4.3	23	3.7	16	3.4	18	4.7	26	3.9	102	4.0
Average respondents and scores		18	4.9	23	3.7	16	3.9	19	5.4	26	4.1	102	4.4

This dimension received among the lowest scores in the index, with an overall score of only 4.4 and individual items that scored even lower. In the national application in the Dominican Republic, for example, except for health workforce analysis of shortages and labor market dynamics, all items scored less than 4, for an overall average of 3.7. Similarly low ratings were given in the subnational application in the DR and in Mali. The only countries that received relatively high scores for the item of effectiveness of health workforce recruitment strategies were Ghana (6.0) and Burkina Faso (5.7). In most countries, respondents complained of maldistribution of the workforce and that there were no or ineffective strategies to retain health workers in rural areas (item #33), and that any benefits to personnel should not only be economic but involve other areas such as accommodation.

### Human Resources Management

The human resources management (HRM) dimension of the index comprises nine items designed to assess whether the country has systems in place to optimize and sustain the health workforce. See Table 7 for the scores obtained for each item and country.

**Table 7:** Number of respondents and their scoring for the Human Resources Management dimension of the HRH Effort Index

Item #	VI. Human Resources Management	Burkina Faso		Dominican Republic—national		Dominican Republic—subnational		Ghana		Mali		Total	
		*N*	Score	*N*	Score	*N*	Score	*N*	Score	*N*	Score	*N*	Score
1.	HRM leadership capacity and availability	19	5.1	25	5.2	16	5.4	19	6.2	25	4.5	104	5.3
2.	Strength of professional associations/councils and their licensing and certification	19	4.7	23	4.0	15	4.5	19	7.9	23	4.6	99	5.1
3.	Existence and availability of human resources manuals/guidelines	19	4.5	23	5.6	16	6.4	19	6.3	23	4.9	100	5.5
4.	Performance management practices	19	4.6	23	5.0	16	5.3	18	5.3	24	4.9	100	5.0
5.	Performance evaluation and rewards	19	5.7	22	4.4	16	4.4	19	5.1	23	4.0	99	4.7
6.	Career development	19	6.5	23	4.0	15	4.4	18	6.2	24	4.2	99	5.1
7.	Health workforce occupational safety and health (OSH) strategy	18	5.2	24	5.4	16	5.3	18	4.1	24	4.2	100	4.8
8.	Nondiscrimination, equal opportunity, and gender equality in the workplace	18	5.6	24	5.2	16	5.8	18	5.1	21	5.1	97	5.4
9.	Assessment of health workforce productivity and quality	18	4.7	24	4.5	16	4.7	19	4.7	24	4.0	101	4.5
Average respondents and scores		19	5.2	23	4.8	16	5.1	19	5.7	23	4.5	99	5.0

While this dimension scores at the mid-point overall, there are some important variations within countries and individual items. For example, Mali consistently scored lower than other countries, especially on the items of performance evaluation and rewards and assessment of health workforce productivity and quality. These two items typically drew among the lowest scores for the dimension. Contrastingly, the item of existence and availability of human resources manuals and guidelines produced average or higher scores in all countries except Burkina Faso. An interesting finding arises in the strength of professional associations or councils and their licensing and certification, which elicited relatively low scores in most countries except in Ghana, where it received among the highest ratings (7.9) observed in the index, which counterbalanced other scores to produce an average of 5.1 for the item.

### Monitoring, Evaluation, and Information Systems

The last dimension—Monitoring, Evaluation, and Information Systems—includes eight items designed to assess the country’s capacity to collect, manage, analyze, and use data related to the health workforce. See Table 8 for scoring of individual items and overall score by country.

**Table 8:** Number of respondents and their scores for the Monitoring, Evaluation, and Information Systems dimension of the HRH Effort Index

Item #	VII. Monitoring, Evaluation, and Information Systems	Burkina Faso		Dominican Republic—national		Dominican Republic—subnational		Ghana		Mali		Total	
		*N*	Score	*N*	Score	*N*	Score	*N*	Score	*N*	Score	*N*	Score
1.	Monitoring and evaluation of national HRH plan	16	5.1	22	4.5	14	5.4	16	4.8	22	5.1	90	5.0
2.	Monitoring and evaluation implementation capacity	16	4.7	23	4.8	15	5.6	16	4.6	24	4.4	94	4.8
3.	Use of data in HRH planning	18	5.2	22	4.4	15	4.6	16	4.9	24	5.3	95	4.9
4.	Staffing and employment information system	19	4.1	21	3.9	14	4.2	16	5.5	24	4.8	94	4.5
5.	Interoperability	16	4.1	22	4.4	12	4.7	15	3.9	19	5.1	84	4.4
6.	National health workforce registry	15	4.8	21	4.6	14	5.4	15	5.1	21	5.8	86	5.1
7.	Health worker licensure and registration system	16	5.3	22	5.0	15	6.4	17	7.6	20	4.6	90	5.8
8.	Information and communications technology infrastructure and capacity	18	3.8	23	4.9	16	5.8	18	4.4	25	5.0	100	4.8
Average respondents and scores		17	4.6	22	4.6	14	5.3	16	5.1	22	5.0	92	4.9

As with other dimensions (Finance, Education, and Training), a few respondents did not complete this dimension, probably reflecting its more specialized nature or difficulty among the categories. Items that scored lower in this dimension included whether the country has a staffing and employment information system (4.5)—particularly in the DR national application (3.9)—and the degree of interoperability of information systems related to HRH such as between payroll and other health management information systems (4.4), which received the lowest score in Ghana (3.9). Burkina Faso produced a low score on the infrastructure and capacity of the information and communications technology (3.8). A respondent there complained that HR software installed in 2014 had yet to prove effective to improve their HR information system. On the other hand, respondents in Ghana gave an extremely high score to their health worker licensure and registration system (7.6), stating that there is a “well structured” system for doctors, nurses, and midwives, which will be extended to other qualified health personnel. This item also scored relatively well in the subnational Dominican Republic application (6.4).

### Overall Scores

**Fig. 1:**
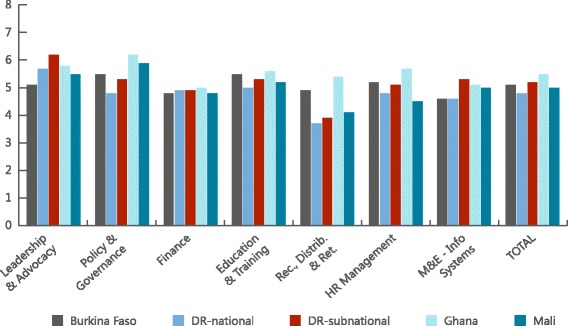
Scores obtained for each dimension and the total overall index score, by country

Figure 1 illustrates the scores obtained for each dimension and the total overall index score, by country. The figure clearly shows that the dimension most in need in these countries is the Recruitment, Distribution, and Retention of the health workforce. This dimension places special emphasis on the deployment of health workers in rural and remote areas.

The next most pressing area of need is financing, where scores struggled to reach the mid-point. Interestingly, the scores were almost the same for all countries. The Monitoring, Evaluation and Information Systems dimension received low marks in Burkina Faso and the Dominican Republic. Only in Mali did the management of HR receive a score that was comparatively lower than the other countries.

By contrast, the areas of Leadership and Advocacy and Policy and Governance displayed among the highest scores, in most countries surpassing the mid-point. The exception was the Dominican Republic, at the national level, which produced a score in this area that was lower than for the finance and education and training dimensions. Scores for other dimensions rested around the mid-point, except for HR Management: the high score in Ghana was close to 6, while the low was 4.5 in Mali.

With the aggregation of scores, it is difficult to see country variations, but for several dimensions the Dominican Republic produced among the lowest scores in the index. On the high end of the scoring, the outlier is Ghana, whose scores are generally at odds with the other countries, perhaps reflecting a combination of the true picture and a sense of pride on progress on HRH issues among its respondents.

## Discussion

The HRH Effort Index can provide new and standardized information on the inputs and processes in the area of HRH. It can be used to take stock of the advances and limitations—nationally as well as subnationally—in dimensions affecting the health workforce, and thus prioritize HRH efforts. In Mali, it also proved a useful tool to convene stakeholders, guide discussions, and arrive at a consensus about health workforce strengthening needs.

Regarding results, one pattern emerged during the analysis. Individual items within dimensions were generally scored with sufficient variation to allow for identification of weaker areas; however, averaging scores almost always produced a modulated dimension score that centered around the mid-point (5). Whether respondents felt inclined to compensate a critical scoring for one item with another that was more generous within the same dimension, or simply that the subjective nature of several questions made it unavoidable to score them moderately cannot be concluded without further analyses and studies. This indicates the need to pay special attention to scores between and within dimensions in each country. Additionally, instructions should clearly reinforce the neutrality of the questionnaire (i.e., intent to assess current status, not find fault) and confidentiality of responses. Finally, sampling strategies should ensure application of the index to the widest and most varied audience possible.

Within the limitations expressed above, the index applications did provide insight into the most critical areas of HRH. The weakest area by far was the deployment and retention of the health workforce in rural and remote areas. The other critical area was lack of financial resources devoted to HRH. This included a variety of factors reflecting insufficient budgetary allocations (e.g., to build capacity of offices, support students to complete preservice education, provide incentives for health workers to remain in underserved locations) as well as financing by external (i.e., international) partners. It was, therefore, surprising that respondents did not tie these deficiencies to the Leadership and Advocacy or Policy and Governance dimensions, which received higher scoring throughout. Whether this indicates lack of critical capacity (or individuals’ fear of being identified through responses), or reflects the ease of assigning a generous score to an item difficult to be measured objectively, remains to be seen.

The index has been applied only in a few countries. As has been done with similar indices, subsequent applications should provide more information about how discriminant and sensitive respondent measurements are. Another alternative is to look for new anchor indicators (e.g., the percentage of the HRH budget coming from domestic sources; the number of policies enforced; actual graduation, vacancy, and retention rates) whose values may help respondents score areas more objectively. Given the relative novelty of HRH indicators in public health, increased efforts and more research are needed to improve the evidence base.

## Conclusions

Despite the limitations of the HRH Effort Index, its testing in two countries and application in five countries, along with comments expressed by respondents, provided valuable information to formulate recommendations for strengthening HRH in these countries and elsewhere:

- *Develop and implement a comprehensive approach to strengthening the health workforce that addresses each of the dimensions assessed in the index.* This process should engage a representative group of HRH stakeholders (e.g., in an HRH observatory or technical working group) that can advocate effectively for HRH and influence decision-makers and funding.
- *Engage preservice education stakeholders to develop approaches to support students from enrollment through employment.* This includes addressing key student needs (e.g., tuition assistance, distance education, gender equity) and factors affecting the critical period between graduation and absorption into the health workforce.
- *Develop strategies to address health workforce shortages and barriers to equitable health workforce distribution.* Countries should focus on developing recruitment and retention strategies that not only increase the overall number of health workers but make postings in hard-to-reach areas of the country more attractive to health workers and/or compulsory for some period of time.
- *Increase capacity to manage and support the health workforce.* Countries should continue investing in developing good decision-making capacity among HRH leaders and managers, and in creating work environments that promote safety, reward good performance, and provide career progression.
- *Improve HRH information systems for collecting, managing, and analyzing health workforce data.* This includes the needed information and communications technology infrastructure and maintenance, and fostering a culture of monitoring and evaluating HRH activities.

The HRH Effort Index should be applied regularly to assess progress. It can also be replicated at subnational levels to view results of decentralized HRH efforts. The index has been translated into French and Spanish, allowing for application in many parts of the world. Additional applications will increase the sample size and allow for meta-analyses, looking for more patterns and correlations, differentials by respondents’ characteristics, and other findings. Additional implementation of the index could also allow for further research into the need for weighting of individual items and dimensions, recombination of items and dimensions, and correlation of the index with output measures (e.g., personnel turn-over rates, client/staff ratios) and others of access, use, coverage, and quality of services, and possibly with selected health outcomes. The index has the potential to become a valuable tool to assist in health systems strengthening.

## Abbreviations

API: AIDS Program Effort Index
DR: Dominican Republic
FP: Family planning
FPE: Family Planning Effort Index
GHWA: Global Health Workforce Alliance
HAF: Human Resources for Health Action Framework
HR: Human resources
HRH: Human resources for health
HRM: Human resources management
MOH: Ministry of Health
SBA: Skilled birth attendance
USAID: United States Agency for International Development
WHO: World Health Organization
